# Identification of *Plasmodium falciparum* proteoforms from liver stage models

**DOI:** 10.1186/s12936-019-3093-3

**Published:** 2020-01-07

**Authors:** Benjamin Winer, Kimberly A. Edgel, Xiaoyan Zou, Julie Sellau, Sri Hadiwidjojo, Lindsey S. Garver, Christin E. McDonough, Neil L. Kelleher, Paul M. Thomas, Eileen Villasante, Alexander Ploss, Vincent R. Gerbasi

**Affiliations:** 1 Naval Medical Research Center, 503 Robert Grant Avenue, Silver Spring, MD 20910 USA; 2 The Henry M Jackson Foundation, 6720A Rockledge Dr., Rockville, MD 20817 USA; 3 Walter Reed Army Institute of Research, 503 Robert Grant Avenue, Silver Spring, MD 20190 USA; 4 Department of Molecular Biology, Princeton University, Washington Road, Princeton, NJ 08544 USA; 5 Northwestern University National Resource for Translational Proteomics, Evanston, IL 60208 USA; 6 Present Address: Department of Molecular Biology and Immunology, Molecular Infection Immunology, Bernhard Nocht Institute for Tropical Medicine, Bernhard-Nocht-Straße 74, 20359 Hamburg, Germany

**Keywords:** Proteomics, Liver stage, Top-down, Vaccine, Antigen, Cell-mediated immunity

## Abstract

**Background:**

Immunization with attenuated malaria sporozoites protects humans from experimental malaria challenge by mosquito bite. Protection in humans is strongly correlated with the production of T cells targeting a heterogeneous population of pre-erythrocyte antigen proteoforms, including liver stage antigens. Currently, few T cell epitopes derived from *Plasmodium falciparum*, the major aetiologic agent of malaria in humans are known.

**Methods:**

In this study both in vitro and in vivo malaria liver stage models were used to sequence host and pathogen proteoforms. Proteoforms from these diverse models were subjected to mild acid elution (of soluble forms), multi-dimensional fractionation, tandem mass spectrometry, and top-down bioinformatics analysis to identify proteoforms in their intact state.

**Results:**

These results identify a group of host and malaria liver stage proteoforms that meet a 5% false discovery rate threshold.

**Conclusions:**

This work provides proof-of-concept for the validity of this mass spectrometry/bioinformatic approach for future studies seeking to reveal malaria liver stage antigens towards vaccine development.

## Background

Nearly half of the world’s population is at risk of contracting malaria. In 2017, there were an estimated 219 million malaria cases and approximately 435,000 deaths [1]. In humans, malaria is caused by *Plasmodium* species, of which *Plasmodium falciparum* and *Plasmodium vivax* are the major contributors to human morbidity and mortality. An effective malaria vaccine would reduce deaths and could accelerate the systematic elimination of malaria. As a result, investigators worldwide have sought to develop malaria vaccines that either block infection altogether, block transmission, or control infection loads at the blood stage [2].

During a *Plasmodium* infection cycle, mosquitoes introduce sporozoites into the skin of their host while taking a blood meal. The sporozoites that enter the blood stream migrate to the liver sinusoid, are thought to traverse Kupffer cells occupying endothelial fenestrae, and translocate through multiple hepatocytes before invading and initiating development in a final human liver cell [3]. After 8–10 days of replication in a parasitophorous vacuole, merozoites are released from their consumed hepatocyte and infect red blood cells. Blood stage merozoites continue to replicate and induce the symptoms of malaria.

Development of a vaccine that targets a portion of the parasite life cycle preceding the blood stage would stop malaria disease symptoms and block transmission of the parasite. Because these vaccines would target the sporozoite or liver stages, they are commonly referred to as “pre-erythrocytic” or “pre-red blood cell” (pre-RBC) vaccines. For the past several decades investigators have focused on pre-erythrocytic vaccines because of the radiation-attenuated sporozoite (RAS) paradigm [4–10]. Inoculation of RAS into humans by either mosquito bite or intravenous delivery protects humans from re-challenge with non-irradiated sporozoites [5, 9, 10]. This long-standing experimental vaccine paradigm suggests that it is possible to design a pre-erythrocyte vaccine that will provide complete sterile protection from malaria infection.

RAS immunization induces CD8+ and CD4+ T cells that kill malaria-infected hepatocytes [11–14]. Priming of antigen-specific effector T-cells by RAS in human and mouse infection models plateaus after the first immunization, [7, 15] suggesting that subsequent homologous RAS boost provides only minor gains in parasite-targeting T cell populations. RAS stimulates CD8+ T cells against liver stages by presenting pre-erythrocyte antigens through MHC Class I molecules on hepatocytes. After RAS sporozoites invade hepatocytes, parasite development stalls [16] resulting in degradation of a heterogeneous population of malaria pre-erythrocyte stage proteins. These are subjected to proteosomal degradation, and peptide cleavage products are subsequently loaded onto MHC class I molecules and presented on the hepatocyte surface. These degraded proteins undergo processing that is poorly understood, although preference for presentation via MHC Class I appears to favor parasite antigens containing a PEXEL domain [17].

Fragmentation of malaria proteins by host or parasite machinery leads to a plethora of proteoform antigens (truncated peptide fragments that no longer resemble the mass of the full-length protein and may contain post-translational modifications), which have previously been inaccessible for characterization. Identification of malaria proteoforms from liver stages would define putative antigens that induce the protective immunity afforded by RAS. Past studies by Tarun and Kappe successfully identified liver stage tryptic peptides from *Plasmodium yoelii* by enriching for fluorescently-labelled parasites [18]. More recently, Sinnis and colleagues characterized *P. berghei* merosome proteins released from HepG2 cells [19]. Discovery of the presented malaria liver stage antigens has remained elusive because malaria is a complex organism expressing > 5000 gene products [20], all of which can ultimately code for multiple different polypeptide species (proteoforms).

Currently, there exists a technological gap in the ability to identify the most commonly presented malaria liver stage antigen proteoform epitopes. This technological gap has left several essential questions in liver stage malaria unexplored. For example, there exists a distinct possibility that non-MHC Class I proteoforms are processed by the host machinery. Second, the segregation of the parasite vacuolar membrane from the host cytoplasm creates a barrier between the malaria proteoforms and host proteases. Hence the degree at which malaria proteoforms are digested and presented relative to host proteoforms has remained uncharacterized.

The aim of this study was to identify malaria proteoforms during the liver stage. By combining a parasite culture system in primary human hepatocytes (PHHs) and chimeric humanized mouse livers with multi-dimensional-protein-identification-technology (MudPIT) [21, 22], and top-down bioinformatics analysis [23], 229 *P. falciparum* proteins and 6185 host proteins were identified at a 5% false discovery rate (FDR). Collectively, these results suggest that direct proteoform sequencing is a viable approach to identify liver stage malaria antigens that can serve as vaccine candidates.

## Methods

### Animal studies

The chimeric mouse studies were performed at Princeton University. Animals were cared for in accordance with the Guide for the Care and Use of Laboratory Animals, and all protocols (number 1930) were approved by the Institutional Animal Care and Use Committees (IACUC). All facilities are accredited by the Association for Assessment and Accreditation of Laboratory Animal Care (AAALAC) International and operate in accordance with the NIH and U.S. Department of Agriculture guidelines and the Animal Welfare Act.

### Engraftment of adult human hepatocytes into FAH−/− NOD Rag1−/− IL2Rγ^NULL^ (FNRG) mice

FNRG mice were generated and transplanted as previously described [24, 25]. Female mice between 6 and 10 weeks of age were injected with approximately 1.0 × 10^6^ cryopreserved adult human hepatocytes. Primary human hepatocytes were obtained from BioIVT (Westbury, NY). FNRG mice were cycled on NTBC (Yecuris Inc, Tualatin OR) supplemented in their water to block the build-up of toxic metabolites. FNRG mice were maintained on amoxicillin chow. Hepatocyte engraftment was monitored by ELISA for human albumin.

### Albumin ELISA for assessment of human hepatocyte engraftment of chimeric mice

Levels of human albumin in mouse serum were quantified by ELISA; 96-well flat-bottomed plates (Nunc, Thermo Fischer Scientific, Witham MA) were coated with goat anti-human albumin antibody (1:500, Bethel) in coating buffer (1.59 g Na_2_CO_3_, 2.93 g NaHCO_3_, 1L dH_2_O, pH = 9.6) for 1 h at 37 °C. The plates were washed four times with wash buffer (0.05% Tween 20 (Sigma Aldrich, St. Luis MO) in 1× PBS) then incubated with superblock buffer (Fisher Scientific, Hampton NH) for 1 h at 37 °C. Plates were washed twice. Human serum albumin (Sigma Aldrich, St. Luis MO) was diluted to 1 µg/mL in sample diluent (10% Superblock, 90% wash buffer), then serial diluted 1:2 in 135 µL sample diluent to establish an albumin standard. Mouse serum (5 µL) was used for a 1:10 serial dilution in 135 µL sample diluent. The coated plates were incubated for 1 h at 37 °C, then washed three times. Mouse anti-human albumin (50 µL, 1:2000 in sample diluent, Abcam, Cambridge, UK) was added and plates were incubated for 2 h at 37 °C. Plates were washed four times and 50 µL of goat anti-mouse-HRP (1:10,000 in sample diluent, LifeTechnologies, Carlsbad, CA) was added and incubated for 1 h at 37 °C. Plates were washed six times. TMB (100 µL) substrate (Sigma Aldrich, St. Luis, MO) was added and the reaction was stopped with 12.5 µL of 2 N H_2_SO_4_. Absorbance was read at 450λ on the BertholdTech TriStar (Bad Wildbad, Germany).

### Infection of human liver chimeric humanized mice

The chimeric FNRG mice were infected with 1 × 10^6^ freshly dissected *P. falciparum* NF54 sporozoites through tail vein injection. Seven days after inoculation mice were sacrificed, chimeric livers were removed, and livers were placed in OCT (optimal cutting temperature) media and immediately frozen at − 80 °C. Mouse liver sections were stained with either anti-CSP Cat #MRA-183A (1:100 BEI resources, Manassas, VA) or anti-*P. falciparum* HSP70 LifeSpan Biosciences, Inc (Seattle, WA) (1:50) followed by anti-mouse secondary antibody (1:200) or anti-rabbit secondary antibody (1:100) with either Hoechst stain (1:2000) or DAPI.

### Extraction of proteoforms from chimeric mouse livers

Sporozoite-infected chimeric mouse livers preserved in OCT media were thawed and washed 30 mL of 1× PBS by centrifugation at 1000×*g* for 5 min. After centrifugation, the supernatant was removed and replaced with 1 mL of 10% Acetic acid. Infected livers were subjected to dounce homogenization. Liver lysates were centrifuged at 5000×*g* for 5 min and the supernatant (containing proteoforms) was harvested. The proteoform-containing eluents were immediately treated with 1 mL of 1 M Tris pH 7.5 to neutralize the acetic acid and stabilize proteoforms. Eluents from infected liver lysates were centrifuged through spin filters with a Microcon 10 kDa mass cut-off (Millipore, Burlington, MA) at 10,000×*g* for 15 min. The retentate was discarded and the flow through was harvested and subjected to desalting over a reverse-phase C8 macrotrap column (Michrom Bioresources, Auburn, CA) and lyophilized via speedvac.

### Culture of primary human hepatocytes (PHHs)

Primary human hepatocytes were cultured as described by Zou et al. [26]. Briefly, primary human donor hepatocytes were purchased from BioIVT, Inc (Baltimore, MD). 200,000 viable hepatocytes from three different human donors were plated per well. The hepatocytes were plated on LabTek^R^ (ThermoFisher, Watham, MA) chamber slides and inoculated with 100,000 *P. falciparum* NF54 freshly dissected sporozoites. Following inoculation hepatocytes were washed every 24 h with 1× phosphate-buffered saline and fresh media was provided. PHH cells were fixed and stained with anti-*P. falciparum* HSP70 LifeSpan Biosciences, Inc (Seattle, WA) (1:50), anti-rabbit secondary antibody (1:100), DAPI, and Evans Blue at 72 and 196 h after inoculation.

### Isolation of proteoforms from infected primary human hepatocytes

Sporozoite-infected hepatocytes were washed in 500 µL of 1× PBS by centrifugation at 1000×*g* for 5 min. After centrifugation the supernatant was removed and replaced with 500 µL of 10% Acetic acid to liberate proteins and protein fragments. Hepatocyte lysates were centrifuged at 1000×*g* for 5 min and the supernatant (containing proteoforms) was harvested. The proteoform-containing eluents were immediately treated with 500 µL of 1 M Tris pH 7.5 to neutralize the acetic acid and stabilize proteoforms. Eluents from infected hepatocytes were centrifuged through spin filters with a Microcon 10 kDa mass cut-off filter (Millipore, Burlington, MA) at 10,000×*g* for 15 min. The retentate was discarded and the flow through was harvested and subjected to desalting over a reverse-phase C8 chromatography column and lyophilized.

### Intact mass spectrometry of proteoforms

The desalted and lyophilized proteoforms were subjected to MudPIT (multi-dimensional-protein-identification-technology) as described previously [27]. Briefly, proteoforms were loaded onto a strong cation exchange column packed in-line with C8 reversed phase chromatography material. The proteoforms were electrosprayed into a ThermoFisher Q Exactive Plus mass spectrometer (ThermoFisher, Bremen, Germany). MS1 resolution was set to a resolution of 70,000. The top 15 ions were selected for fragmentation. MS2 resolution for fragmented proteoform spectra was set to a resolution of 17,500. Proteoforms were sequenced using a dynamic exclusion setting duration of 30 s to identify lower abundance proteoforms.

### Database searches and identification of proteoforms

For monoculture samples, a human *P. falciparum* (strain NF54) UniProt concatenated database was generated and loaded onto the Galaxy server and searched using TD Portal. For humanized mouse samples, a human-mouse *P. falciparum* (strain NF54) UniProt concatenated database was generated and loaded onto the Galaxy server and searched using TD Portal. For both human and chimeric mouse samples proteoforms were reported at a 5% FDR cut-off. Host and parasite proteoforms with spectral matches above and below the 5% FDR are included in Additional file 2: Table S1 and Additional file 3: Table S2.

### Quantification of schizont size and number of merozoites per schizont

Image processing was performed using Fiji and Nikon elements software (Nikon, Minato, Tokyo, Japan). The area quantification tool was utilized to determine schizont size. The dot identification tool was utilized for merozoite quantification by using it in the DAPI channel and limiting it to the schizont area.

### Statistical analysis of schizonts and merozoites

Statistical analysis was performed using Graphpad Prism Software (Graphpad, La Jolla, CA). A nonparametric t test was performed. P values less than 0.01 were considered statistically significant.

## Results

### Model of truncated proteoforms generated from malaria-infected hepatocytes

**Fig. 1:**
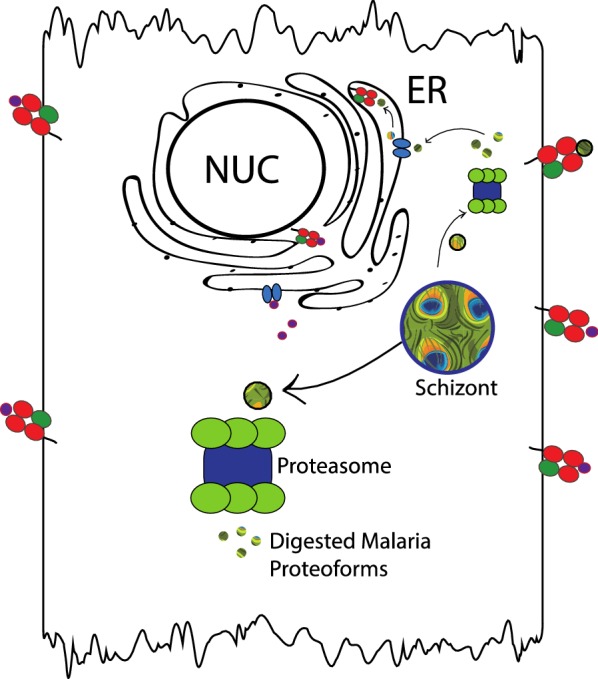
Model of peptide processing in LS schizonts. Schizont proteoforms are processed through the proteasome and presented on MHC Class I through the ER or degraded as non-MHC Class I fragment peptides during schizogony

While several laboratories have reported gains in malaria liver stage model development, these studies have yet to explore the repertoire of host and parasite proteoforms generated during schizont maturation. Given past reports from human and animal models of live sporozoite challenge, it is clear that attenuated sporozoites can induce a population of CD8+ and CD4+ T cells targeting liver stage antigens [7]. The generation of these T cell populations has formed the basis for a model in which hepatocytes present malaria antigens on MHC class I. Also possible, but largely unexplored, is the idea that developing schizont proteoforms are digested by host or parasite proteases in a manner that does not resemble MHC Class I peptides (Fig. 1). These non-MHC Class I proteoforms could exist as random fragments with no potential for interaction with histocompatibility receptors or these fragments could be on-path for additional digestion and subsequent MHC Class I presentation (Fig. 1).

A typical bottom up mass spectrometry approach to identify malaria proteoforms from liver stage models would digest host and parasite fragments with trypsin prior to mass spectrometry analysis. Digestion of the host and malaria proteoforms prior to analysis obfuscates the structure of the proteoform from the liver tissue. To identify malaria polypeptides in their intact form from malaria liver stage models requires an unbiased approach capable of matching candidate mass spectra from non-digested host and parasite proteoforms. To capture native host and parasite proteoforms from liver stage models, this study utilized a top down mass spectrometry approach.

### Characterization of *Plasmodium falciparum* liver stage models

**Fig. 2:**
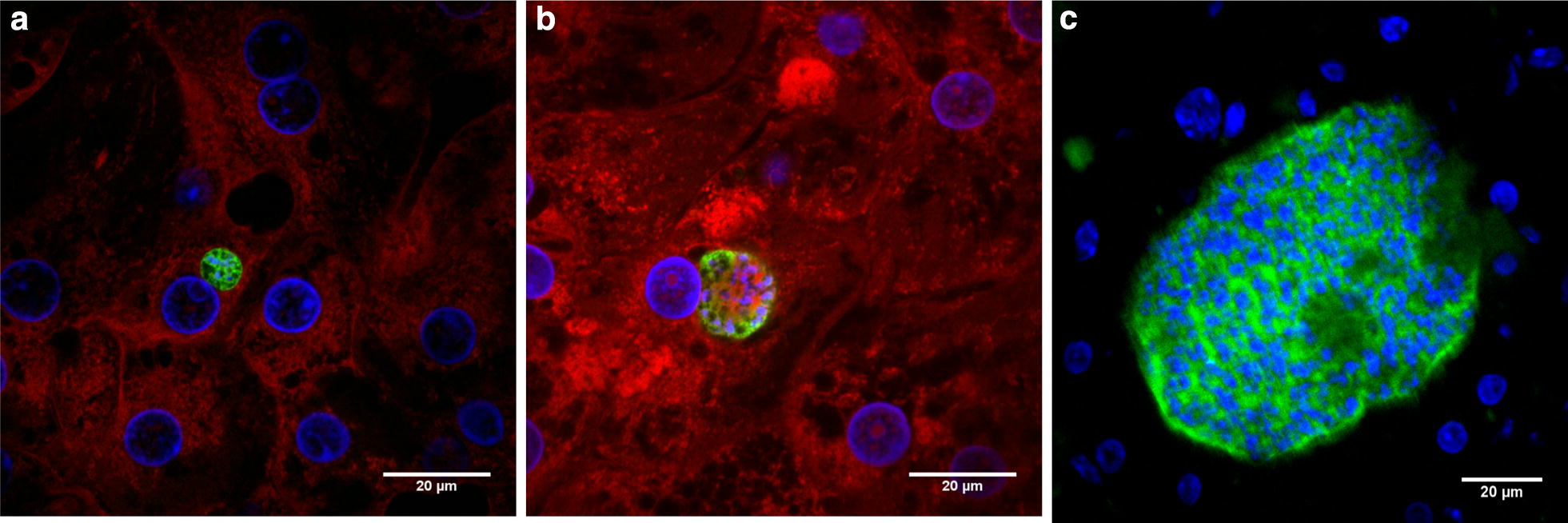
NF54 *P. falciparum* malaria liver stage schizonts. **a** Primary human hepatocyte monoculture cells infected with *P. falciparum* NF54 malaria sporozoites were stained 72 h after inoculation with HSP70 antibodies, DAPI, and Evans Blue. **b** The identical experiment was performed as in (**a**) except cells were fixed and stained after 192 h (8 days). **c** LS schizonts from humanized mice infected with NF54 sporozoites were fixed and stained 7 days after sporozoite inoculation with Hoechst and anti-CSP antibodies

**Fig. 3:**
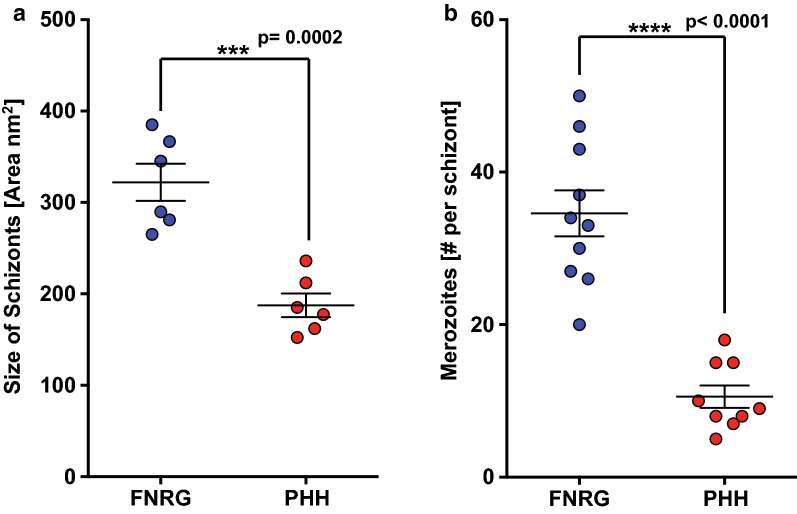
Quantification of schizont size and number of merozoites in PHHs from FNRG mice and in vitro monocultures. **a** Schizonts from P.f. infected FNRG mice and from monocultured PHHs were imaged with size determined. **b** The number of merozoite progeny in monocultured human hepatocytes and FRNG mice were determined

Towards identification of liver stage malaria proteoforms this study first analysed parasite growth characteristics in state-of-the-art experimental liver stage models including PHHs grown in vitro and human liver chimeric (FNRG) mice [26, 28–30]. Primary human hepatocytes were transplanted into FNRG mice which after several weeks became highly engrafted as seen by ~ 1 × 10^4^ µg/mL human albumin level in the serum of transplanted animals (Additional file 1: Figure S1). The chimeric FNRG mice were subsequently injected with *P. falciparum* NF54 sporozoites to form liver stage parasites. PHHs were inoculated with *P. falciparum* NF54 sporozoites and subsequently stained at different time points with anti-*P. falciparum*-HSP70 antibodies. Seventy-two hours after sporozoite inoculation of PHHs, parasite schizonts were apparent and smaller than the hepatocyte nucleus (Fig. 2a). Eight days later the parasite schizonts exceeded the size of the hepatocyte nucleus, but merozoite formation was disorganized and the overall growth of the in vitro schizont was stunted (Fig. 2b). In contrast, infected hepatocytes from human liver chimeric mice inoculated with *P. falciparum* NF54 sporozoites via tail vein injection and stained with anti-*P. falciparum*-CSP had large, organized schizonts filled with merozoites that dwarfed the size of the hepatocyte nucleus 7 days after inoculation (Fig. 2c). The size of schizonts in human liver chimeric FNRG mice and PHH cultures were quantified (Fig. 3a). Schizonts from engrafted FNRG mouse livers were statistically larger (p = 0.0002) ranging from 260 to 385 nm^2^, while schizonts from PHH mono-cultures were 162 nm^2^ to 212 nm^2^ in size. In addition, there were statistically more numerous merozoites (p < 0.0001) per schizont in FNRG humanized mice ranging from 20 to 50 versus 5 to 18 in PHH mono-cultures (Fig. 3b). These results suggest that while the in vitro PHH model supports schizont development during the first few days after sporozoite inoculation, the chimeric humanized mouse liver model is a more supportive conduit for late liver stage development. This could be due to the fact that the latter human hepatocytes have a transcriptional profile more similar to what is observed in the human liver because they are in the three-dimensional context of the murine liver.

### Characteristics of host and parasite proteoforms from liver stage models

Mild acid elution (10% acetic acid) and molecular weight spin columns were used to extract low molecular weight proteoforms from intact chimeric human/mouse liver tissue and PHHs infected with *P. falciparum* NF54 sporozoites. The use of 10% acetic acid will result in only acid-soluble proteoforms being identified as a possible limitation of the approach. Due to the aforementioned developmental capacity of the chimeric human/mouse model, livers were harvested 7 days after infection with *P. falciparum* NF54 sporozoites. PHHs were harvested 96 h after infection prior to the apparent disruption in schizont development observed in Fig. 2b. Soluble, acid-extracted proteoforms were subjected to multi-dimensional chromatography and tandem mass spectrometry followed by bioinformatics analysis using TDPortal and TDViewer [23]. Proteoform MS/MS spectra were searched against a concatenated tripartite Human-Mouse-NF54 database and scrambled decoy database to identify spectral matches from humanized mouse experiments whereas proteoform spectra from human hepatocyte monocultures were searched against a concatenated Human-NF54 database with a matched scrambled decoy database. Top-down bioinformatics tools were employed to: (1) identify fragmented proteoform species isolated in their native state and (2) to accurately control the FDR associated with mass spectrometry identification of proteins and proteoforms as described recently by Leduc et al. [23].

**Fig. 4:**
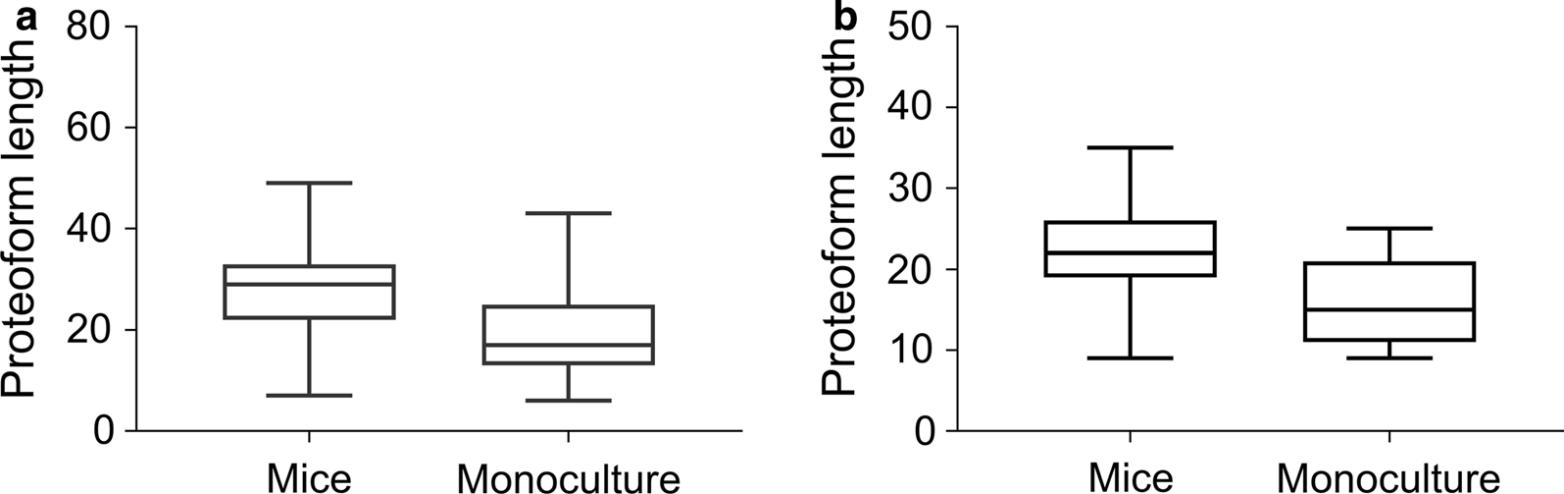
Proteoform length from liver stage models. **a** Whisker boxplot of host proteoform sequence length (in amino acids) from humanized mice (mice) or primary human hepatocytes (monoculture), **b** malaria proteoform sequence length (in amino acids) The first quartile of proteoform length is indicated by the lower error bar. The third quartile is indicated by the upper error bar. The median proteoform length is indicated by a horizontal line in the box

Using TDPortal at a 5% proteoform FDR cutoff, a total of 5343 unique proteins were identified from three different infected human chimeric mouse livers. Host and parasite proteoform spectra identified from these studies that did not pass the 5% FDR cut-off are included in Additional file 2: Table S1 but were not analysed here. In Additional file 2: Table S1 and Additional file 3: Table S2 a global Q-value is included that is the equivalent of an individual FDR value for each proteoform [31]. From three biological replicate human primary hepatocyte (monoculture) samples a total of 1339 total unique proteins were identified. The length distribution of host (Fig. 4a) and parasite proteoforms (Fig. 4b) was analysed from infected chimeric human mouse livers and PHHs. Results showed a mean amino acid length of 24.4 (standard deviation of 11.86), and a median length of 29 for host proteoforms from humanized mouse livers and a mean length of 26.8 (standard deviation of 25.3), and a median length of 17 from primary human hepatocytes. Parasite proteoforms had a mean length of 22.5 amino acids (standard deviation of 6.0), and a median length of 22 from humanized mouse livers and a mean length of 16.4 (standard deviation of 5.9) amino acids, and a median length of 15 in primary human hepatocytes. These results suggest that both host and parasite proteoforms from liver stage models are within the expected size range of MHC Class I peptides (8–12 amino acids), MHC Class II peptides (12–31 amino acids), and randomly degraded proteoform fragments.

### Identification of host and parasite proteins sequenced across biological replicates

**Fig. 5:**
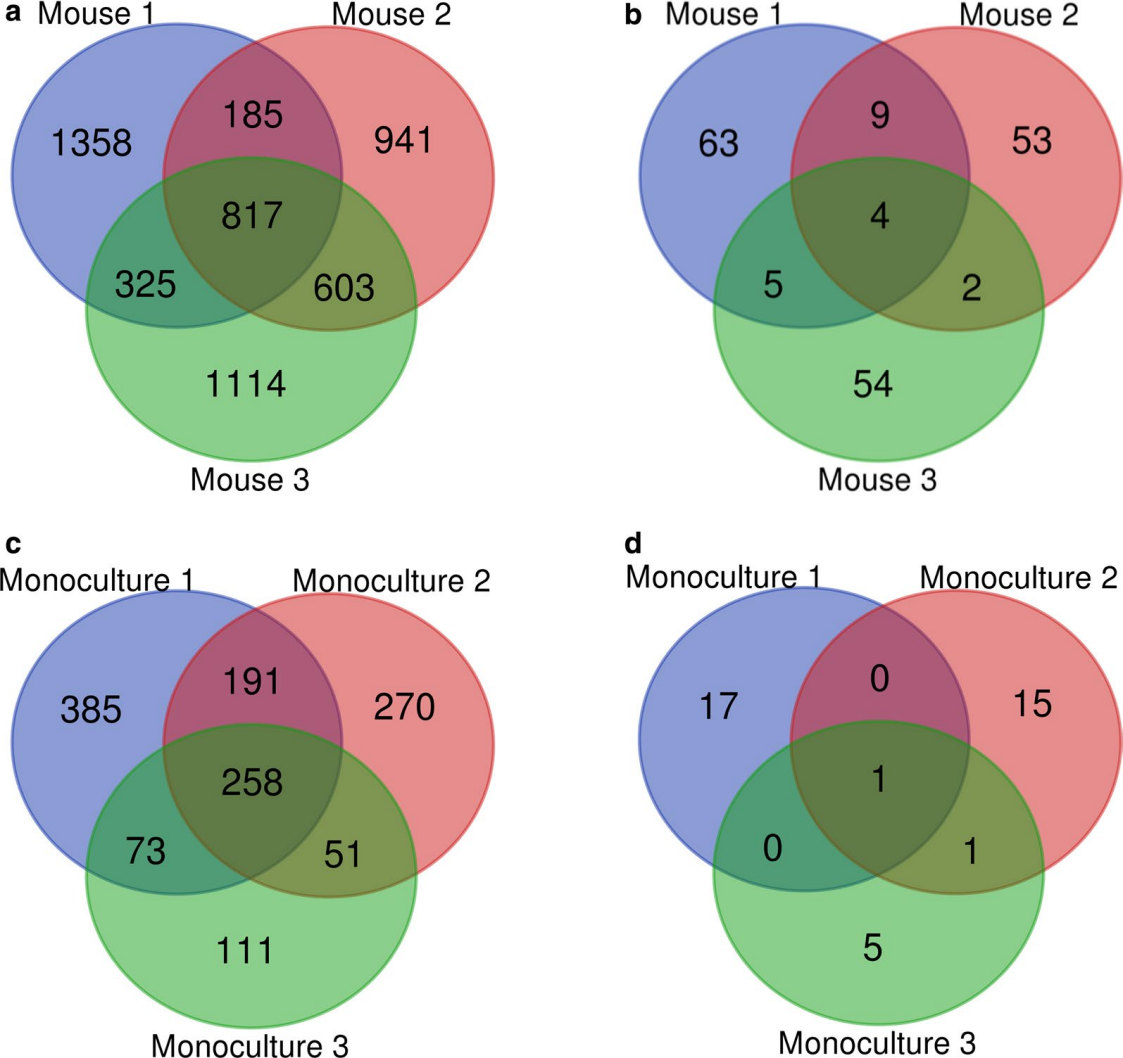
Shared proteins identified between different biological replicate samples. **a** Venn diagram of shared host proteins from humanized mouse livers infected with NF54 sporozoites. **b** Venn diagram of shared malaria proteins from humanized mouse livers infected with NF54 sporozoites. **c** Venn diagram of shared host proteins from primary human hepatocytes infected with NF54 sporozoites. **d** Venn diagram of shared malaria proteins from primary human hepatocytes infected with NF54 sporozoites

To identify host and *P. falciparum* proteins that were sequenced across different biological replicates of infected humanized mouse livers and infected primary human hepatocytes sample sets were analysed using venn diagrams. Among three humanized infected mouse liver samples 5343 total host proteins (Fig. 5a) and 190 total *P. falciparum* proteins were identified (Fig. 5b). Conserved proteins from infected chimeric mouse livers, (representing those that were sequenced in two or more samples), numbered 1930 (36% of total) among host proteins (Fig. 5a) and 20 (10.5% of total) among *P. falciparum* proteins (Fig. 5b). Among infected primary human hepatocytes, a total of 1339 total Human proteins (Fig. 5c), 39 total *P. falciparum* proteins (Fig. 5d), 573 (42% of total) conserved Human proteins (Fig. 5c), and two (5% of total) conserved *P. falciparum* proteins were identified across biological replicates (Fig. 5d). The four proteins identified among all infected humanized mouse livers were W7KN90 (an uncharacterized protein), W7K8P5 (26S proteasome regulatory subunit), W7JYB7 (Actin-2), and W7K9G1 (DNA polymerase epsilon catalytic subunit A) (Table 1). Among infected primary human hepatocyte samples a single protein (W7K7Q9), encoding an uncharacterized protein, was sequenced in all three biological replicates (Table 2).

**Table 1:** Uniprot accession number, amino acid sequence, sequence length, C-score, presence or absence in sporozoites, presence or absence in blood stages, PEXEL domain, IEDB immunogenicity score, and PlasmoDB (3D7) identification number of malaria proteoforms identified from > 1 biological replicate of humanized mouse livers infected with NF54 sporozoites

Protein name	Peptide sequence	Peptide length	C-score	Present in sporozoites (yes/no)	Present in blood stages (yes/no)	PEXEL domain	Immunogenicity score	PlasmoDB
Uncharacterized protein	LESQKKMKIFLLSYNIFQKMDNPVD	25	58.7	No	Yes	No	− 1.14019	PF3D7_0210500
26S proteasome regulatory subunit	YYINKKEIDKLIEFTTSNE	19	36.8	Yes	Yes	Yes	− 0.08337	PF3D7_1402300
Actin-2	WITKEEYEDSGPSIVHRK	18	1059.7	Yes	Yes	No	0.0321	PF3D7_1412500
DNA polymerase epsilon catalytic subunit A	FILNIDAYKWVERDSYLPNGSRTLKSVC	28	128.08	Yes	Yes	Yes	− 0.17081	PF3D7_0630300
Uncharacterized protein	RFTHILSIHIIE	12	222.1	Yes	Yes	No	0.35298	PF3D7_1108900
Uncharacterized protein	SYLEKHLKIEDLDECIKKKRND	22	106.3	No	Yes	No	− 0.45115	PF3D7_0622100
Uncharacterized protein	MSNILYVSILLLLYFLDLYS	20	34.2	No	Yes	No	0.19188	PF3D7_1452400
Uncharacterized protein	MVPKNILFIFVILFHLIGYLKN	22	222.7	No	No	No	0.56696	PF3D7_1336700
LCCL domain-containing protein	TKLFFINYAFVIIFSFNLFVKC	22	85	Yes	Yes	No	0.86824	PF3D7_1455800
Uncharacterized protein	TYVKIHEKLGSDFYNILHANII	22	17.8	No	Yes	No	0.09123	PF3D7_0612400
Uncharacterized protein	EKQKLVEEKQKLVDEKQNLIDE	22	56.7	Yes	Yes	No	− 0.62876	PF3D7_0903600
MSP7-like protein	IKGLIFYLFICFFVFFVHA	19	183.8	Yes	Yes	No	0.96628	PF3D7_1334600
Uncharacterized protein	PLYYEHKFSDLKIQQSYTP	19	109.2	Yes	Yes	No	− 0.56439	PF3D7_0728100
p1/s1 nuclease	SNIFVIFCSLLILIFTKRCSG	21	54.6	No	Yes	No	0.30636	PF3D7_1411900
Merozoite surface protein 8	VFKSSYIFFFLFLVILYFNNVVEG	24	84.4	Yes	Yes	No	0.55183	PF3D7_0502400
Uncharacterized protein	MPLLFFILLIIYFHLIVC	18	124.6	No	Yes	No	0.84834	PF3D7_0934000
Leucine–tRNA ligase	KKRSYVNWSNELRCVISNDELRNEINL	27	3	Yes	No	No	0.37112	PF3D7_0828200
SUN domain-containing protein	MIWWFLISVNFLFFLIKS	18	82.9	Yes	Yes	No	0.70747	PF3D7_1439300
Acetyl-CoA synthetase	KLKHIEIKK	9	139.5	Yes	Yes	No	0.17272	PF3D7_0627800
Uncharacterized protein	ISLFYSIYICLEKLCKK	17	47.9	Yes	Yes	Yes	− 0.15264	PF3D7_0809600

**Table 2:** Uniprot accession number, amino acid sequence, sequence length, C-score, presence or absence in sporozoites, presence or absence in blood stages, PEXEL domain, IEDB immunogenicity score, and PlasmoDB (3D7) identification number of malaria proteoforms identified from > 1 biological replicate of primary human hepatocytes infected with NF54 sporozoites

Accession #	Protein name	Peptide sequence	Peptide length	C-score	Present in sporozoites	Present in blood stages (yes/no)	PEXEL domain	Immunogenicity score	PlasmoDB
W7K7Q9_PLAFO	Uncharacterized protein	YNKIIFQLRN	10	274.6507446	Yes	No	No	0.21936	PF3D7_0820800
W7K7I5_PLAFO	Translocon component PTEX150	RIIILALLIVCTIINYYCA	19	24.10918248	Yes	Yes	No	0.55272	PF3D7_1436300

Conserved *P. falciparum* proteoforms sequenced from infected chimeric mouse livers ranged in length from 9 to 29 amino acids. The C-score, indicating relative level of proteoform characterization among conserved proteoforms ranged from 3 to 1059 (Table 1) in the infected humanized mouse samples. The C-score utilizes native proteoform mass data (precursor ion information) and proteoform fragmentation data to pair the best match against the target proteomes (Human and/or Mouse, and *P. falciparum* NF54 in this case) [31]. C-scores > 40 indicate extensive characterization, while C-scores between 3 and 40 are identified but only partially characterized [31]. Conserved *P. falciparum* proteoforms sequenced from infected primary human hepatocytes ranged in length from 10 to 19 amino acids with a C-score ranging from 24 to 274 (Table 2).

Infected chimeric humanized mouse livers contained large mature liver forms. Consistent with the late liver stage two different merozoite surface proteoforms derived from MSP8 and MSP7-like proteins were identified (Table 1) from the infected mouse liver samples. Overall, a strong representation (90%) of proteins expressed at blood stages from the chimeric liver samples was observed whereas 65% of proteins were expressed at sporozoite stages. Fifteen-percent of proteins identified from the chimeric mouse livers contained PEXEL domains.

Among proteins identified in the primary human monoculture samples- both are expressed in sporozoite stages. Proteoforms derived from PTEX150 are expressed in both sporozoite and liver stages. While none of the monoculture proteins contained PEXEL domains, PTEX150 is a structural component of the translocation system that moves parasite molecules from the PVM to the host cytoplasm [32, 33].

Among the proteoforms sequenced from the monoculture and chimeric humanized mouse samples the Immune epitope database and analysis resource (IEDB) MHC Class I immunogenicity tool was used to test which proteoform sequences had the highest likelihood of inducing T cell responses [34]. Proteoforms with the highest IEDB immunogenicity score were derived from the MSP7-like protein identified in humanized mouse samples containing an IEDB score of 0.966. Consistently sequenced proteoforms from chimeric humanized mouse samples had IEDB scores ranging from (− 1.14 to 0.966) (Table 1). Monoculture IEDB scores were moderate and ranged from (0.219 to 0.552) (Table 2).

## Discussion

The goal of this study was to test the technical feasibility of sequencing proteoform signatures from human liver cells infected with *P. falciparum*. Results of this study suggest that combining MudPIT and top-down bioinformatics approaches can distinguish host proteoforms from parasite proteoforms and identify liver stage polypeptides near the mass range of MHC Class I and MHC Class II restricted epitopes.

After sporozoites enter the hepatocyte cytoplasm, they form a parasite vacuolar membrane that interfaces with the host autophagy system [35–37]. Parasites have designed a system to escape this endogenous cytoplasmic immunity that involves disrupting autophagy and lysosome interactions with the parasitophorous vacuole membrane (PVM) [38]. Specifically, the parasite tubovesicular network can sequester host factors that damage the PVM [37]. Liver stage schizonts that increase in size and ultimately succeed in the developmental process do not have autophagy and lysosomal markers associated with the PVM [37]. These past studies suggest that the parasite has evolved mechanisms to evade degradation by the host cytoplasmic immune response.

While the PVM can function as a protective barrier for parasite development, exchange of parasite material (proteins, lipids, and nucleic acids) between the liver stage PVM and host cytoplasm remains an unexplored possibility. In this study, mass spectrometry was used to identify host and malaria proteoforms from liver stages. Because an enrichment mechanism is lacking to specifically harvest schizont-containing hepatocytes, samples in this study likely contained uninfected cells, infected cells with aborted development, and infected hepatocytes with vegetative schizonts. Hence proteoforms sequenced in these studies could be derived from intact or aborted schizonts. Additionally, experiments using chimeric mouse livers that were frozen, thawed, and homogenized could generate degraded proteoforms that reflect degradation during sample preparation rather than parasite metabolic activity. Currently, these two forms cannot be distinguished.

Infection of 200,000 hepatocytes with 100,000 *P. falciparum* sporozoites typically results in 0.1–0.2% of the cells infected with mature schizonts after 96 h post-inoculation [26]. Thus, a majority of hepatocytes inoculated with sporozoites do not mount productive infections. MudPIT sequencing from infected hepatocytes resulted in the identification of both Human and *P. falciparum* proteins at a ratio of 34:1 (Human: *P. falciparum*). The *P. falciparum* species represent approximately 2.9% of the total protein population. Given that so few hepatocytes are infected with mature schizonts after 96 h (post-sporozoite inoculation), this result is surprising in that one might expect the ratio to be nearly 1000:1 (Human: *P. falciparum* proteins). Additionally, each schizont represents only a portion of the total hepatocyte mass. However, there are at least two likely reasons for this result. First, the initial parasite to hepatocyte ratio is 1:2 (during inoculation). In theory, a perfect infection, where each sporozoite gave rise to one schizont would result in 50% of hepatocytes harbouring schizonts. Inoculation only results in 0.1–0.2% of cells containing schizonts [26]. Hence, a majority of parasites must invade hepatocytes and then subsequently abort their development between 0 and 96 h post-inoculation. Proteins from sporozoites that fail to develop in hepatocytes are most likely degraded. Therefore, *P. falciparum* proteoform identifications from this study are likely derived from both mature schizonts and sporozoites that failed to develop. A second reason for observing an unexpectedly high number of *P. falciparum* peptides from these hepatocytes is mostly technical. During these experiments, the mass spectrometer isolates and fragments the most abundant peptides. After the first round of peptide isolation and fragmentation, the instrument selects for a lower abundance ion and continues to select for progressively lower abundance ions in a mode known as dynamic exclusion. Importantly, because the instrument in these studies employed dynamic exclusion mode, the number of *P. falciparum* proteoform identifications was enhanced.

While these studies are encouraging and provide a proof-of-concept for sequencing proteoforms from liver stages, recent improvements in mass spectrometry instrumentation could enhance similar studies aimed at identifying malaria liver stage antigens. For example, the development of Tribrid instruments, containing three mass analysers in tandem are capable of identifying more spectra and are compatible with gas-phase separation techniques (such as high-field asymmetric ion mobility spectrometry) (FAIMS). From a complex mixture of polypeptides, FAIMS can select proteoforms of interest that have specific size, charge, or shape characteristics [39–41]. This study and others that aim to isolate small polypeptides (similar in size to MHC Class I), often perform biochemical (often separation by MW) fractionation prior to mass spectrometry analysis [42–44]. During fractionation much of the low molecular weight polypeptides are lost. In future experiments utilization of FAIMS could circumvent the need for size separation prior to mass spectrometry and instead use gas-phase fractionation to sequence malaria and self-peptides from liver stages. This experimental modification should increase the number and depth of proteoforms identified from malaria liver stages by reducing sample loss experienced during off-line size fractionation and selecting for smaller proteoforms using the FAIMS device.

Numerous antigen discovery efforts have led to the identification of pre-erythrocyte antigens that induce sterile immunity from malaria challenge. Some of these antigens function either partially or entirely through CD8+ T cells. For example, the circumsporozoite protein (CSP) is highly expressed in sporozoites and early liver stages, induces antibodies [3, 30, 45–49], induces immunodominant CD8+ T cells that confer protective immunity in naïve mice [50], and protects humans from experimental malaria challenge [51]. Despite the strong expression and assumed MHC Class I presentation of CSP at liver stages, this study failed to detect these antigens as dominantly presented peptides. That MHC Class I restricted peptides from CSP are presented to CD8+ T cells during the course of malaria infection suggests that our approach has limitations in sensitivity. Additionally, *P. falciparum* CSP contains a repeating amino acid pattern of NANPN that accounts for 40% of the predicted protein sequence. Fragment ions from cleavage adjacent to proline ions dominate tandem mass spectra. The dominance of proline within CSP fragments could reduce the number of fragment ion matches resulting in a failure to match CSP spectra to the *P. falciparum* proteome. As a second possibility, malaria liver stage samples for these studies were taken at timepoints ≥ 96 h after sporozoite inoculation, where CSP expression could wane as the parasite transitions to late liver stages.

While our analysis failed to detect circumsporozoite proteoforms, both humanized mouse livers and hepatocyte monocultures detected sporozoite proteoforms (Additional file 2: Table S1 and Additional file 3: Table S2). Consistent with the recent characterization of *P. berghei* merosomes by Shears et al. [19], MSP7-like and MSP-8 proteoforms were observed in mature liver stage forms isolated from humanized mouse samples but not monoculture samples (Additional file 3: Table S2). These results support our observation that chimeric humanized mice support development of mature liver stages. The truncated schizont development observed in the monoculture system suggests that it should be considered for use in future studies aiming to characterize early liver stages whereas the chimeric humanized mouse model shows potential to be useful for analysis of early and late liver stages.

## Conclusions

Attenuated sporozoites provide an effective mechanism to prime immune responses, and ultimately induce protection against genetically homologous or heterologous parasite challenge [7]. Currently, approaches to prime and boost with sporozoites results in a plateau of T cell populations targeting parasites [7, 15]. Priming with a diverse population of genetically attenuated sporozoites followed by boost with an orthogonal immunization mechanism might enhance the breadth and depth of protection beyond what has been achieved in past human malaria challenge trials. Here, an approach was described to identify liver stage proteoforms from in vivo and in vitro parasite culture systems. These experiments provide an experimental proof-of-concept to identify liver stage antigens that serve as orthogonal immunization targets following attenuated sporozoite inoculation.

## Supplementary information

**Additional file 1: Figure S1.** FNRG mice are highly engrafted with primary human hepatocytes. FNRG mice were transplanted with cryopreserved primary human hepatocytes. Mice were bled every few weeks, serum was isolated, and a human albumin ELISA was run to assess engraftment levels.

**Additional file 2: Table S1.** Host and malaria proteoforms identified from chimeric humanized mouse livers or primary human hepatocytes (No FDR).

**Additional file 3: Table S2.** Host and malaria proteoforms identified from chimeric humanized mouse livers or primary human hepatocytes (5% FDR).

## Data Availability

The authors agree to make any and all raw data files available upon request.

## Abbreviations

RBC: red blood cell
RAS: radiation attenuated sporozoites
MHC: major histocompatibility complex
MudPIT: multi-dimensional-protein-identification-technology
PHH: primary human hepatocytes
FDR: false discovery rate
ELISA: enzyme-linked-immunosorbent-assay
NTBC: 2-(2-nitro-4-trifluoro-methylbenzoyl)-1,3-cyclohexanedione
FNRG: FAH−/− NOD Rag1−/− IL2R**γ**NULL mice
TMB: 3,3′,5,5′-tetramethylbenzidine
CSP: circumsporozoite protein
FAIMS: high-field-asymmetric-ion-mobility-spectrometry
MW: molecular weight
