# Supplementary figures and images for: Identification of Plasmodium falciparum proteoforms from liver stage models
Source: Malar J. 2020 Jan 7;19:10. doi: 10.1186/s12936-019-3093-3 (PMC6947969; doi:10.1186/s12936-019-3093-3)

## Slide 1
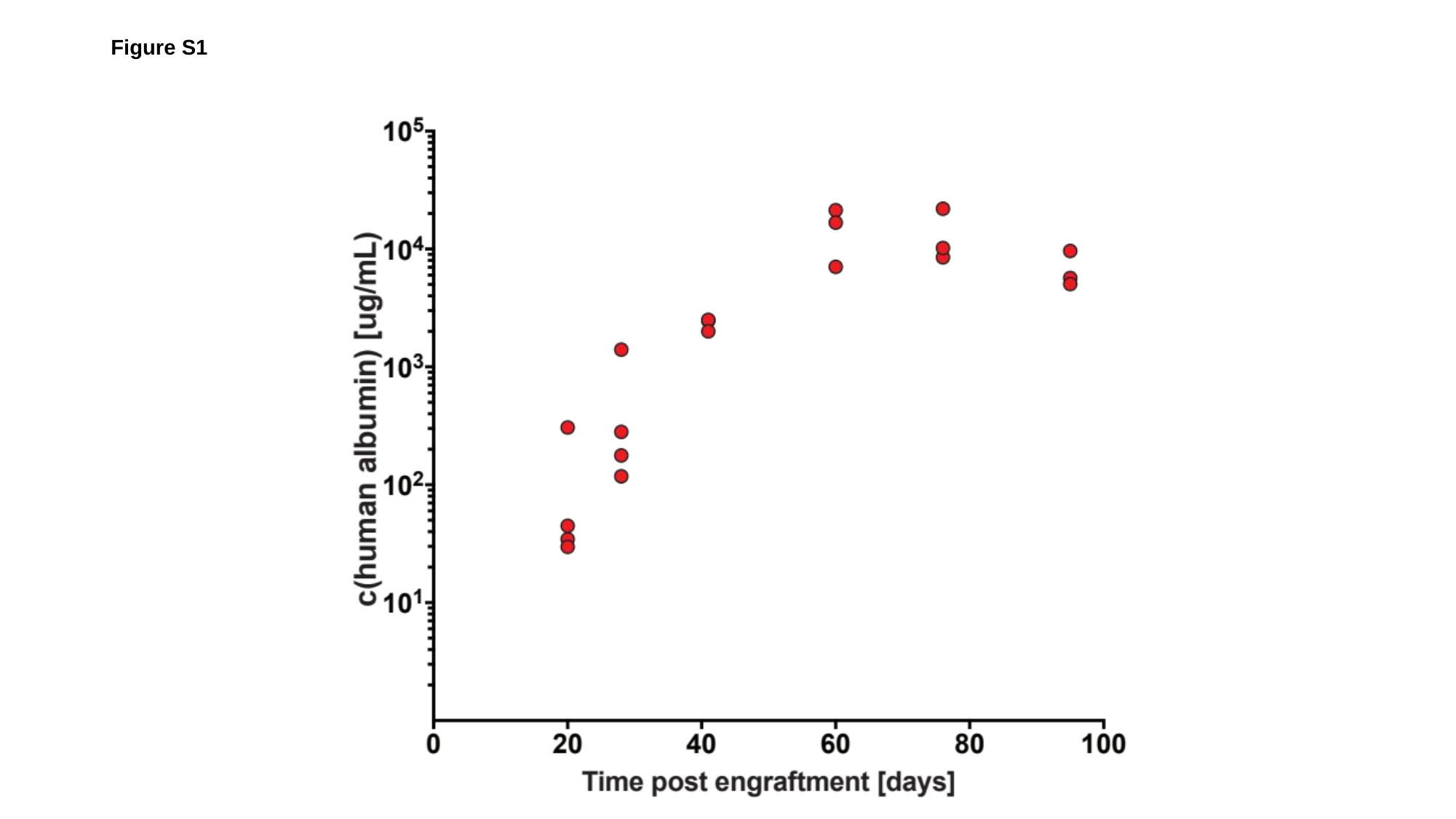

# Figure S1

Supplement: Supplementary file 1 — Additional file 1: Figure S1. FNRG mice are highly engrafted with primary human hepatocytes. FNRG mice were transplanted with cryopreserved primary human hepatocytes. Mice were bled every few weeks, serum was isolated, and a human albumin ELISA was run to assess engraftment levels. [file 12936_2019_3093_MOESM1_ESM.pptx]
